# Supplementary material for: UNC-16/JIP3 and UNC-76/FEZ1 limit the density of mitochondria in C. elegans neurons by maintaining the balance of anterograde and retrograde mitochondrial transport
Source: Sci Rep. 2018 Jun 12;8:8938. doi: 10.1038/s41598-018-27211-9 (PMC5997755; doi:10.1038/s41598-018-27211-9)
Supplement: Supplementary file 1 — Supplementary material [file 41598_2018_27211_MOESM1_ESM.docx]

**SUPPLEMENTARY INFORMATION**

**UNC-16/JIP3 and UNC-76/FEZ1 limit the density of mitochondria in *C. elegans* neurons by maintaining the balance of anterograde and retrograde mitochondrial transport**

Author list:

Guruprasada Reddy Sure^˜, †, #^, Anusheela Chatterjee^‡, #^, Nikhil Mishra^‡^, Vidur Sabharwal ^‡^, Swathi Devireddy^†^, Anjali Awasthi^†, §^, Swetha Mohan^†^, Sandhya P. Koushika^‡, *^

^˜^ Sastra University, Tirumalaisamudram, Tamil Nadu, India 613401

^†^ NCBS-TIFR, Bangalore, Karnataka, India 560065

^‡^ DBS-TIFR, Mumbai, Maharashtra, India 400005

^§^ BITS Pilani, Pilani, Rajasthan, India 333031

^#^ The authors have contributed equally to the manuscript.

^*^ Corresponding author

**
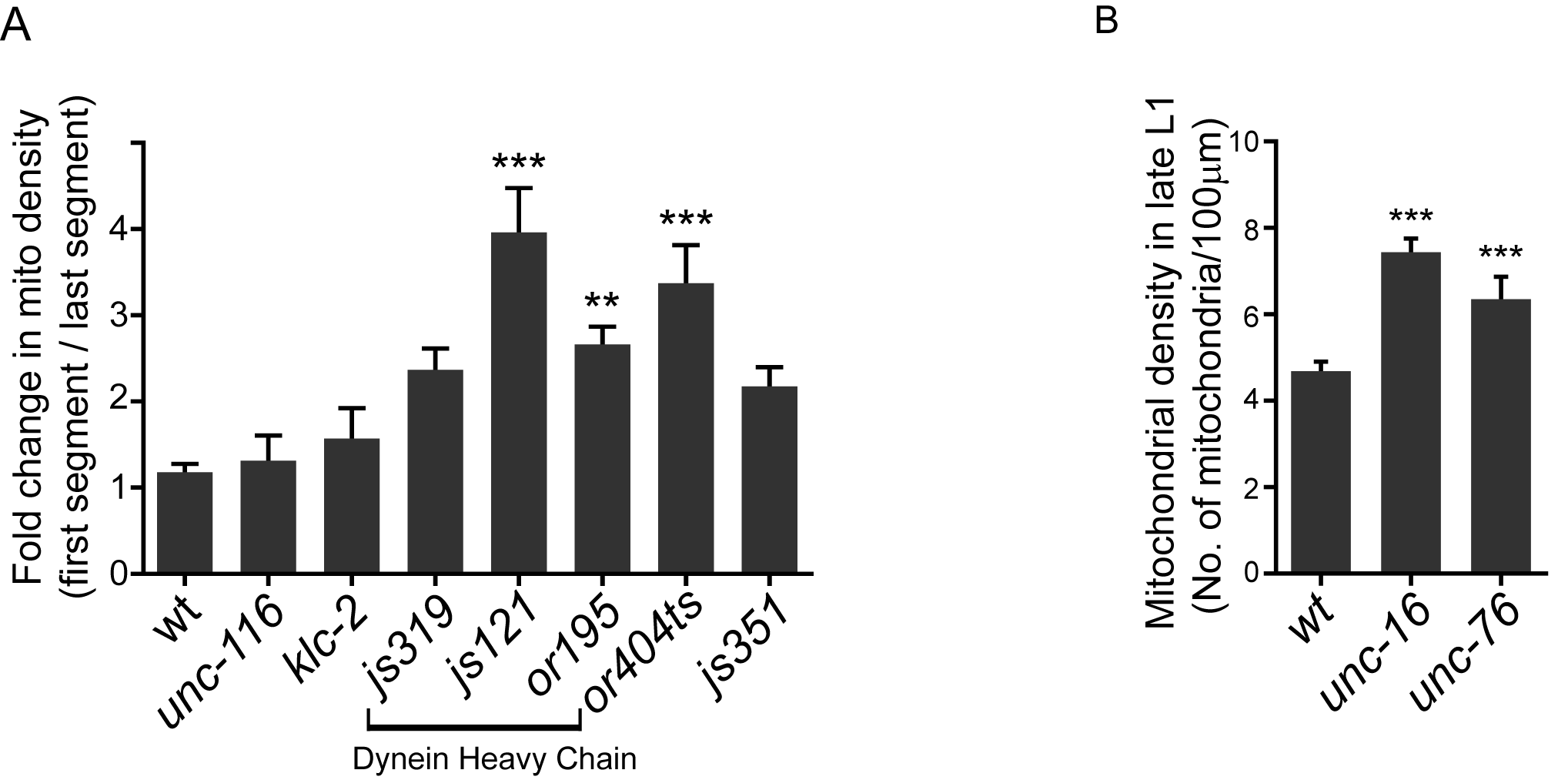
**

**Figure S1:** **(A)** Fold change in mitochondrial density between the first and last segment in wild type (*jsIs609*), *unc-116(e2310), klc-2(km11), dhc-1(js319), dhc-1(js121), dhc-1(or195), dnc-1(or404ts)* grown at 22^O^C and *dli-1(js351).* n≥7 worms. All comparisons made to wild type. Comparisons that are not significant are not listed. **(B)** Average density of mitochondria in late L1 animals of wild type (*jsIs609*), *unc-16(tb109)* and *unc-76(n2397)*. n≥19 worms. Data represented as Mean ± SEM. Statistical tests one-way ANOVA with Bonferonni multiple comparisons correction, p value **<0.01, ***<0.001.

**
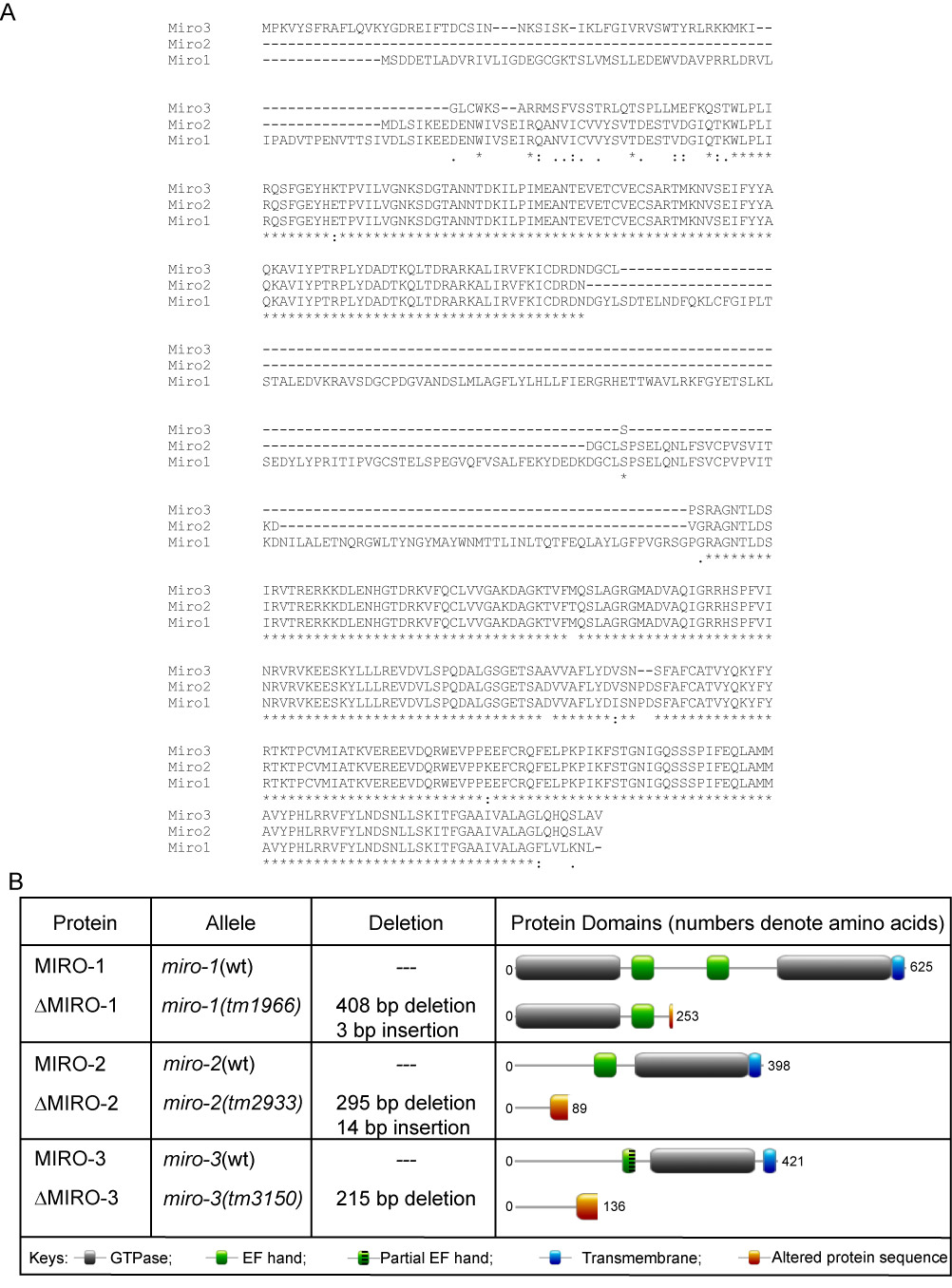
**

**Figure S2: (A)** Alignment of *C. elegans* MIRO-1, MIRO-2 and MIRO-3 proteins. **(B)** Protein domains in wild type and predicted protein domains in mutant *miro* alleles.

**
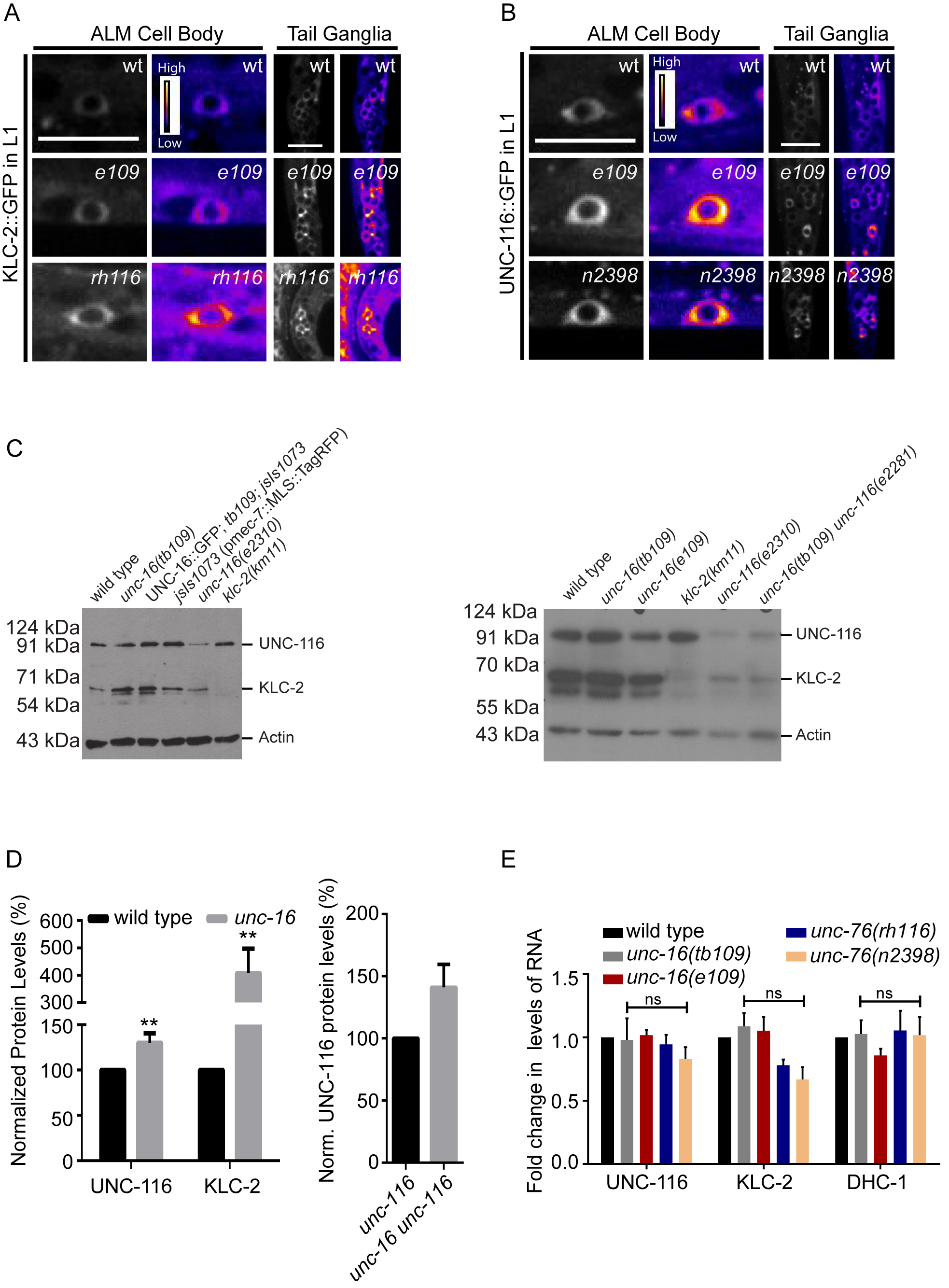
**

**Figure S3:** Levels of Kinesin-1 protein and RNA in *unc-16* and *unc-76* mutants.

**(A)** Representative images of KLC-2::GFP in ALM cell body and tail ganglia in L1 animals of wild type, *unc-16(e109), unc-76(rh116)*. **(B)** Representative images of UNC-116::GFP in ALM cell body and tail ganglia in L1 animals of wild type, *unc-16(e109), unc-76(n2398)*. **(C)** Full length blots showing levels of Kinesin-1 protein subunits. The observation with the following genotypes- (i) UNC-16::GFP; *unc-16(tb109); jsIs1073*, (ii) *jsIs1073* and (iii) *unc-16(e109)-* have not been analyzed for this study. These full length blots relate to Figure 3H. **(D)** Average normalized (norm.) intensity of Western blot signal showing elevated levels of both Kinesin-1 protein subunits in whole worm lysates prepared from *unc-16(tb109)* animals and UNC-116 protein levels in whole worm lysates of *unc-116(e2310)* and *unc-16(tb109) unc-116(e2281)*. *Unc-116(e2310)* and *unc-116(e2281)* are the same allele (see Table S1). All comparisons made to wild type in first graph and *unc-116(e2310)* in the second graph. Mann-Whitney test, n≥3 independent protein extracts, western blots from each extract done in duplicates. **p<0.01. Second graph p=0.06 **(E)** Fold change in mRNA levels of *unc-116*, *klc-2* and *dhc-1* in *unc-16* and *unc-76* mutants. All comparisons are not significant (ns) and made to wild type using one-way Anova with Bonferroni multiple comparison correction.

**Table S1:** Strain List

| Genotype | Reference |
| --- | --- |
| *N2* | 57 |
| *jsIs609: Is[_p_mec-7::mls::gfp]* | 30 |
| *jsIs1073: Is[_p_mec-7::mls::rfp]* | 48 |
| *unc-116(e2310)* | *unc-116(e2310)* and *unc-116(e2281)* are the same allele 32 |
| *unc-116(rh24sb79)* | 33 |
| *unc-116(f122)* | 34 |
| *klc-1(ok2609)* | International *C. elegans* Gene Knockout Consortium 35 |
| *klc-2(km11)* | 21 |
| *klc-2(km28)* | 21 |
| *unc-16(tb109)* | 45 |
| *unc-16(e109)* | 44 |
| *unc-16(n730)* | 44 |
| *unc-16(ce451)* | 24 |
| *unc-76(n2397)* | 27 |
| *unc-76(n2398)* | 27 |
| *unc-76(rh116)* | 27 |
| *unc-76(e911)* | 27, 46 |
| *unc-14(ju56)* | 21 |
| *unc-14(e57)* | 47 |
| *dhc-1(or195)* | 37 |
| *dhc-1(js319)* | 36 |
| *dhc-1(js121)* | 36 |
| *dli-1(js351)* | 36 |
| *dnc-1(or404ts)* | 36 |
| *miro-1(tm1966)* | NBRP |
| *miro-2(tm2933)* | NBRP |
| *miro-3(tm3150)* | NBRP |
| *tbIs224* | We integrated rescuing extrachromosomal arrays- *Ex[_p_unc-116::unc-116::gfp]* 48 |
| *tbIs235* | We integrated rescuing extrachromosomal arrays- *Ex[_P_klc-2::klc-2::gfp]* 48 |
